# *Neisseria meningitidis* Has Two Independent Modes of Recognizing Its Human Receptor CEACAM1

**DOI:** 10.1371/journal.pone.0014609

**Published:** 2011-01-27

**Authors:** Katharina Kuespert, Alexandra Roth, Christof R. Hauck

**Affiliations:** Lehrstuhl für Zellbiologie, Universität Konstanz, Konstanz, Germany; University of Hyderabad, India

## Abstract

**Background:**

Several human-restricted Gram-negative bacteria exploit carcinoembryonic antigen-related cell adhesion molecules (CEACAMs) for host colonization. For example, *Neisseria meningitidis* engages these human receptors via outer membrane proteins of the colony opacity-associated (Opa) protein family triggering internalization into non-phagocytic cells.

**Principal Findings:**

We report that a non-opaque strain of *N. meningitidis* selectively interacts with CEACAM1, but not other CEACAM family members. Using functional assays of bacterial adhesion and internalisation, microscopic analysis, and a panel of CEACAM1 deletion mutants we demonstrate that the engagement of CEACAM1 by non-opaque meningococci occurs in a manner distinct from Opa protein-mediated association. In particular, the amino-terminal domain of CEACAM1 is necessary, but not sufficient for Opa protein-independent binding, which requires multiple extracellular domains of the human receptor in a cellular context. Knock-down of CEACAM1 interferes with binding to lung epithelial cells, whereas chemical or pharmacological disruption of host protein glycosylation does not abrogate CEACAM1 recognition by non-opaque meningococci. The previously characterized meningococcal invasins NadA or Opc do not operate in a CEACAM1-dependent manner.

**Conclusions:**

The results demonstrate a mechanistically distinct, Opa protein-independent interaction between *N. meningitidis* and human CEACAM1. Our functional investigations suggest the presence of a second CEACAM1-binding invasin on the meningococcal surface that associates with the protein backbone and not the carbohydrate structures of CEACAM1. The redundancy in meningococcal CEACAM1-binding factors further highlights the important role of CEACAM recognition in the biology of this human-adapted pathogen.

## Introduction

The genus *Neisseria* contains two human-specific pathogens, *Neisseria gonorrhoeae* and *Neisseria meningitidis*, which occupy different host niches and cause two distinct diseases. Whereas *Neisseria gonorrhoeae* is the causative agent of gonorrhea and primarily infects the urogenital tract causing localized inflammation, *Neisseria meningitidis* is a frequent commensal of the upper respiratory tract, which can cause life-threatening invasive infections, such as septicaemia and meningitis [1], [2]. To cause disease, meningococci need to traverse the mucosal barrier and enter into the bloodstream. There, the bacteria can multiply rapidly, as a polysaccharide capsule and sialylation of lipooligosaccharide renders them resistant against complement-mediated killing [3]. Furthermore, *N. meningitidis* has a propensity to tightly interact with endothelial cells and to cross the blood-brain barrier, resulting in fulminant meningococcal meningitis [4]. Clearly, colonization of the mucosal epithelium is the first step for causing disease, followed by invasion, intracellular persistence and transcytosis [5]. Known meningococcal factors, which promote adhesion to epithelial cells and presumably play a role in colonization are type IV pili, App (adhesion and penetration protein) [6], [7], MspA (meningococcal serin protease A) [8], NhhA (Neisserial hia/hsf homologue) [9] and HrpA [10]. Additionally, meningococci express a panel of proteins that not only mediate adhesion, but also promote invasion into host cells, such as colony opacity associated (Opa) proteins, Opc and NadA [11]. NadA belongs to the oligomeric coiled-coil (Oca) family of adhesins and seems to be expressed primarily in hyper-virulent *Neisseria meningitidis* lineages, but not in *Neisseria gonorrhoeae* [12]. The cellular receptor for NadA is still unknown – however, there is evidence that the receptor is of protein nature [13]. In contrast to NadA, Opc and Opa proteins belong to class 5 outer membrane proteins. Opc is a phase variable protein, and, though the *opc* gene is found also in gonococci, the protein is only expressed by meningococci [14]. Opc associates with several host molecules including extracellular matrix proteins, integrins and heparansulfate proteoglycans [15], [16], [17]. Unlike Opc, Opa proteins are expressed in most meningococcal and gonococcal isolates. Whereas the meningococcal genome encodes up to 4 distinct Opa proteins, gonococci harbour up to 11 copies of *opa* genes [18]. Expression of Opa proteins is subject to phase variation due to a RecA-independent insertion or deletion of pentanucleotide repeats within the leader peptide coding sequence, which leads to translational reading frame shifts in the constitutively transcribed *opa* genes [19]. In natural settings, phase variation of individual Opa proteins results in a heterogenous population of bacteria expressing none, one or multiple Opa proteins. Upon culture on agar plates, colonies expressing distinct Opa proteins can be differentiated by their phenotype. Besides a few Opa protein variants that recognize cell surface expressed heparansulphate proteoglycans (Opa_HSPG_) [20], [21], most Opa proteins of diverse strains of *Neisseria meningitidis* and *Neisseria gonorrhoeae* recognize one or more members of the carcinoembryonic antigen-related cell adhesion molecule (CEACAM) family (Opa_CEA_) [22], [23], [24]. In particular, CEACAM1, CEACAM3, CEA (the product of the *CEACAM5* gene) as well as CEACAM6 have been reported to bind to neisserial Opa_CEA_ proteins, and to mediate internalization of the pathogens [25], [26]. In this regard, the molecular mechanism of CEACAM3-mediated uptake has been studied in great detail and relies on tyrosine based sequence motifs in the cytoplasmic domain of CEACAM3 [27], [28]. Expression of CEACAM3 is restricted to human granulocytes and enables these immune effector cells to phagocytose and eliminate CEACAM-binding microbes [28], [29], [30]. In contrast to the granulocyte-restricted CEACAM3, CEACAM1 has a broad tissue distribution and is expressed on hematopoetic cells, endothelial cells and epithelial cells [31], [32]. CEACAM1 has attracted particular interest as a homophilic and heterophilic cell-cell adhesion molecule, that is involved in several important cellular activities, such as immune modulation, insulin homeostasis, neoangiogenesis, tissue homeostasis, and tumor progression [33]. This receptor is expressed in several isoforms due to alternative splicing of the *CEACAM1* transcript. The major isoforms are CEACAM1-4L and CEACAM1-4S that possess an extracellular aminoterminal Ig_V_-like domain (N), followed by three Ig_C2_-like domains (A1, B, A2) and a transmembrane domain. CEACAM1-4L and CEACAM1-4S differ in their cytoplasmic domain with CEACAM1-4S having only about ten amino acid residues in the cytoplasm, whereas the long isoform (CEACAM1-4L) has a 71 – 74 residue long cytoplasmic tail. Interestingly, both the long and the short isoform of CEACAM1 internalize bacteria with similar efficiency via a membrane microdomain-dependent uptake mechanism [34]. Though CEACAM1 is a highly glycosylated protein, CEACAM1 binding by neisserial Opa_CEA_ proteins is a direct protein-protein interaction that occurs at the non-glycosylated CC'FG-face of the aminoterminal, Ig_V_-like domain. Therefore, the CEACAM1 aminoterminal, Ig_V_-like domain is necessary and sufficient for binding of Opa_CEA_ protein-expressing gonococci and meningococci [35]. On the bacterial side it has been shown, that the two hypervariable extracellular regions of Opa_CEA_ proteins are responsible for CEACAM recognition [36]. However, no clear consensus has emerged, which amino acid sequence determines the adhesive properties of neisserial Opa_CEA_ proteins. Nevertheless, Opa_CEA_ proteins appear as the only neisserial invasins described so far, which bind to CEACAM1.

In the present study we investigated the interaction of *Neisseria meningitidis* strain MC58 with CEACAMs. Surprisingly, we observed that a variant of *Neisseria meningitidis* MC58, which was selected for the absence of Opa protein expression, was still able to specifically interact with CEACAM1. Similar to the binding of Opa_CEA_ protein-expressing meningococci, the interaction with non-opaque *N. meningitidis* was independent of post-translational carbohydrate modification of the receptor. However, the aminoterminal domain of CEACAM1 was necessary, but not sufficient for this association. Heterologous expression of several known meningococcal invasins did not result in CEACAM1-mediated uptake. Therefore, our data suggest that *Neisseria meningitidis* MC58 possesses a novel, uncharacterized invasin, which mediates CEACAM1-interaction.

## Materials and Methods

### Nessierial strains and growth conditions

Opa_CEA_ protein-expressing, non-encapsulated *Neisseria meningitidis* strain MC58 (ΔSiaD, ΔlgtA) (Nm Opa_CEA_) was obtained from Matthias Frosch (Institut für Hygiene und Mikrobiologie, Universität Würzburg, Germany). The Opa protein-negative derivative of *Neisseria meningitidis* MC58 (Nm Opa-) was isolated from Nm Opa_CEA_ by visual screening for a non-opaque phenotype using a binocular microscope. Opa_52_ protein-expressing, non-piliated *Neisseria gonorrhoeae* strain MS11 (Ngo Opa_CEA_) and non-opaque, non-piliated *N. gonorrhoeae* MS11 (Ngo Opa-) were kindly provided by Thomas F. Meyer (Max-Planck-Institut für Infektionsbiologie, Berlin, Germany). Both, *Neisseria meningitidis* and *N. gonorrhoeae* were grown as described before [35] on GC agar plates (Difco BRL, Paisley, UK) supplemented with vitamins at 37°C, 5% CO_2_. For infection, over-night grown bacteria were taken from GC agar plates, suspended in PBS, and colony forming units (cfu) were estimated by OD_550_ readings according to a standard curve.

### Epithelial and endothelial cell lines

Human embryonic kidney epithelial 293T cells (293 cells; ACC-635, German collection of microorganisms and cell cultures, DSMZ, Braunschweig, Germany) were cultured in Dulbecco's modified Eagle's medium (DMEM) containing 10% calf serum. African green monkey kidney cells (COS cells) and chinese hamster ovary cells (CHO K1 cells), as well as stably transfected COS-CEACAM1 and CHO K1-CEACAM1 cells, were obtained from T.F. Meyer (MPI für Infektionsbiologie, Berlin, Germany) and cultured in RPMI containing 10% fetal calf serum. A549 epithelial cells (human alveolar lung adenocarcinoma cells; ATCC CCL87) were cultured in DMEM containing 10% fetal calf serum. Human brain microvascular endothelial cells (HBMEC) [37] were grown in endothelial cell medium (PAA, Pasching, Austria) supplemented with L-glutamine. Cells were grown at 37°C in 5% CO_2_ and subcultured every 2–3 days.

### Recombinant DNA constructs

Mammalian expression plasmids encoding cDNA of human CEACAM3, CEA, CEACAM6, CEACAM7 or CEACAM8 have been described previously [30]. HA-tagged versions of CEACAM1, CEACAM1 lacking the Ig_C2_-like domains A1, B, A2 (CEACAM1-N) and CEACAM1 lacking the complete cytoplasmic domain (CEACAM1-ΔCT) were described previously [34], [38]. CEACAM1-NA1B-variant and CEACAM1-NA1-variant were generated from the HA-tagged version of CEACAM1 by SOEing PCR [39]. For CEACAM1-NA1B-variant, cDNA for NA1B domains was amplified by PCR with the primers CEACAM1-IF-sense (5′-GAAGTTATCAGTCGACACCATGGGGCACCTCTCAGCCCC-3′) and C1-Chim-NA1B-TM-anti (5′-TACGTTCAGCATGATGGGTGTGGTCCTGTTGCAGC-3′). The transmembrane together with the cytoplasmic domain of CEACAM1 was amplified with primers C1-Chim-NA1B-TM-sense (5′-GCTGCAACAGGACCACACCCATCATGCTGAACGTA -3′) and HA-stop-CEACAM-IF-anti (5′-ATGGTCTAGAAAGCTTTATGCAGCGTAATCTGGAACGTCATATGG-3′). For CEACAM1-NA1-variant, cDNA for NA1 domains was amplified using primers CEACAM-IF-sense and C1-Chim-NA1-TM-anti (5′-TACGTTCAGCATGATGGGGTCACTGCGGTTCGCAC-3′). The transmembrane and the cytoplasmic domain of CEACAM1 was amplified with the primers C1-Chim-NA1-TM-sense (5′-GTGCGAACCGCAGTGACCCCATCATGCTGAACGTA-3′) and HA-stop-CEACAM-IF-anti. The two resulting fragments from each construct were combined and served as templates for SOEing PCR with primers CEACAM1-IF-sense and HA-stop-CEACAM-IF-anti. The CEACAM8-N/1-chimera was generated by SOEing PCR using CEACAM1 and CEACAM8 as template, respectively. The aminoterminal domain of CEACAM8 was amplified by PCR with the primers CEA8-IF-sense (5′-GAAGTTATCAGTCGACACCATGGGGCCCATCTCAGCC-3′) and CEA8/1-Chimera-anti (5′-GGGCTTGGGCAGCTCCGGATGTACGCTGAACTGG-3′). The extracellular Ig_C2_, the transmembrane and the cytoplasmic domains of CEACAM1 were amplified by PCR with the primers CEA8/1-Chimera-sense (5′-TTCAGCGTACATCCGGAGCTGCCCAAGCCCTCC -3′) and HA-stop-CEACAM-IF. The combined fragments served as templates for the SOEing PCR as above. The cDNAs resulting from the SOEing PCRs were cloned into pDNR-CMV using the In-Fusion PCR Cloning Kit (Clonetech, Mountain View, CA).

The secreted CEACAM1 construct (sCEACAM1-N) encoding the amino-terminal domain of CEACAM1 fused to GFP was described by us [35]. The secreted CEACAM1 construct encoding all extracellular domains of CEACAM1 (sCEACAM1) was generated by PCR with primers CEACAM1-IF-sense and CEA1-secret-prot-IF-anti 5′-ATGGTCTAGAAAGCTTGGGTCGCTTTGGTTCTTACTGATTGG-3′ using pcDNA CEACAM1-HA as a template. The same template was used to generate sCEACAM1-NA1B by amplification with primers CEACAM1-IF-sense and CEA1-B-Dom-IF-anti 5′-ATGGTCTAGAAAGCTTGGTGTGGTCCTGTTGCAGC-3′. The amplified cDNAs of sCEACAM1 and sCEACAM1-NA1B were cloned into pDNR-Dual using the In-Fusion PCR Cloning Kit and transferred from pDNR-dual into pLPS3′EGFP by Cre-mediated recombination (Creator System, Clontech, Mountain View, CA) allowing eukaryotic expression of secreted, GFP-tagged proteins.

### Expression of meningococcal proteins in *E. coli*

The Opa_CEA_- and the Opc-expressing *Escherichia coli* strains were described previously [40], [41] and kindly provided by T.F. Meyer and Mark Achtman, respectively (Max-Planck Institut für Infektionsbiologie, Berlin, Germany). In order to express NadA, the *nadA* gene was amplified by PCR from chromosomal DNA of *Neisseria meningitidis* MC58 using the primers NadA-MC58-sense (5′-CGGATCCCATGGGCAAACACTTTCCATCC-3′, NcoI site) and NadA-MC58-anti (5′-CCCGCTCGAGTTACCACTCGTAATTGACGCC-3′, XhoI site). The PCR product was digested with NcoI/XhoI and cloned in pET28a (Novagen).

In order to express Opa proteins, *opa*-genes (*opa1* (ORF NMB0442), *opa2* (ORF NMB0926), *opa3* (ORF NMB1465), *opa4* (ORF NMB1636)) were amplified by PCR from chromosomal DNA of *Neisseria meningitidis* MC58 and cloned in pET28a. In order to suppress phase variation, the cloning strategy from Kupsch et al. [40] was performed. Accordingly, *opa1* and *opa2* were amplified using primers Opa-MC58-mitte-sense (5′-ATCGCTTCTATTTAGCTCTTTATTGTTCAGTTCCCTACTCTTCAGCTCCGCAGCGCAGGCGGCAACTGA-3′) and Opa1/2-MC58-HindIII-anti (5′-GGTCAAAGCTTTCAGAAGCGGTAGCG-3′). The PCR product served as a template for a second PCR using the primers OpaMC58(pET28)-NcoI-sense (5′-GGCGCCCATGGAACCAGCCCCCAAAAAACCTTCTCTCCTGTTCTCATCGCTTCTATTTAGCTCTTTA-3′) and Opa1/2-MC58-HindIII-anti. *Opa3* and *opa4* were amplified using the primers Opa-MC58-mitte-sense and Opa3/4-MC58-HindIII-anti (5′-GGTCAAAGCTTTCAGAAGTGGTAGCGCAT-3′) followed by second amplification using the primers OpaMC58(pET)-NcoI-sense and Opa3/4-MC58-HindIII-anti. The product of the second PCRs from *opa1*, *opa2*, *opa3* and *opa4* were digested with NcoI and HindIII and cloned in pET28a (Novagen). Differentiation of *opa1* and *opa2*, or *opa3* and *opa4* was achieved by sequencing. The pET28a vectors encoding NadA or Opa proteins were transformed in *E. coli* BL21 (DE3; Novagen), which was induced for protein expression by IPTG. All *E. coli* strains were cultured at 37°C in Luria-Bertani (LB) supplemented with appropriate antibiotics.

### Generation of a lentiviral vector encoding CEACAM1 shRNA

The plasmids pLKO.1 and plasmid pLKO.1 shControl [42] were maintained in *E. coli* STBL4 (Invitrogen, Carlsbad, CA). Using the algorithm AA(N_19_) (available online at http://jura.wi.mit.edu/bioc/siRNAext/) we identified sequences that could silence expression of human CEACAM1. According to this prediction, primers shCEACAM1-sense 5′-CCGGAATTGTAGGATATGCAATAGGCTCGAGCCTATTGCATATCCTACAATTTTTTTG-3′ and shCEACAM1-anti 5′-AATTCAAAAAAATTGTAGGATATGCAATAGGCTCGAGCCTATTGCATATCCTACAATT-3′ were synthesized, annealed and cloned into the Agel and EcoRI site of pLKO.1 generating pLKO.1 shCEACAM1. The correct insertion of the shRNA cassette was verified by sequencing. Production of infectious lentiviral particles and transduction of cells was performed as described previously [43]. Stably transduced cells were selected for 1 week in medium containing 1 µg/ml puromycin.

### Generation of CEACAM1-encoding lentiviral particles

To generate a CEACAM1-GFP encoding lentiviral vector, human CEACAM1-4L was amplified from plasmid pcDNA3.1 CEACAM1-4L-HA [38] with the forward primer CEACAM1-Nhe_sense 5′-GAACTGCTAGCACCATGGGGCACCTCTCAG-3′ and reverse primer CEACAM1-AgeI_reverse 5′-GCTAGACCGGTATGTCATAGGGATACTGC -3′. The resulting fragment was cloned in the NheI and AgeI restriction sites of lentiviral vector pLL3.7 [44] resulting in an in-frame fusion between CEACAM1 and the GFP coding sequence. Production of infectious lentiviral particles encoding CEACAM1-GFP or GFP and transduction of cells was performed as described previously [43].

### Antibodies and reagents

Monoclonal anti-Opa and Opc antibodies (clone 4B12/C11 and clone B306, respectively) were obtained from Mark Achtman (Max-Planck Institut für Infektionsbiologie, Berlin, Germany). Monoclonal antibody against CEACAMs (anti-CEACAM; clone IH4Fc; recognizing CEACAM1, CEACAM3, CEA, and CEACAM6) was purchased from AL-Immunotools (Friesoythe, Germany) and used for immunofluorescence staining and FACS analysis. Monoclonal antibodies against CEACAMs (clone D14HD11; recognizing CEACAM1, CEACAM3, CEA, and CEACAM6) or CEACAM1-CEA (clone 4/3/17 recognizing CEACAM1 and CEA) were purchased from Genovac (Freiburg, Germany) and used for Western Blotting or FACS analysis, respectively. Monoclonal antibody against the HA-tag (anti-HA; clone 12CA5) or against tubulin (clone E7) were purified from hybridoma cell supernatants. Rabbit antiserum against *N. gonorrhoeae* and *N. meningitidis* (IG-511) was custom-made by Immunoglobe (Himmelstadt, Germany). Rabbit polyclonal anti-GFP antibody was custom generated and purified (Animal Research Facility, Universität Konstanz, Germany). Tunicamycin was obtained from Sigma-Aldrich (Steinheim, Germany), Cy2-labeled concanavalinA (ConA-Cy2) was purchased from Invitrogen (Karlsruhe, Germany).

### Transfection of cells, cell lysis, and Western Blot

293 cells were transfected by calcium phosphate precipitation using 4 µg of appropriate cDNA in each case. The transfection efficiency ranged between 30 and 40% as reported [30] and transfected 293 cells were employed in experiments 48 h after transfection. Cell lysis and Western blotting were performed as described [45]. Similar amounts of cell lysate were used for each sample analysed by SDS-PAGE and Western Blotting.

### Gentamicin protection assay

Gentamicin protection assays were conducted as described [28]. Cells were seeded at 4×10^5^ cells/well in 24 well plates coated with fibronectin (4 µg/ml). A multiplicity of infection (MOI) of 40 bacteria per cell was routinely used, and after 2 hour of infection, extracellular bacteria were killed by 45 min incubation in 50 µg/ml gentamicin in DMEM. Then, cells were lysed with 1% saponin in PBS for 15 min. The samples were diluted with PBS, and the number of viable bacteria was determined by plating suitable dilutions on GC agar. For inhibition studies, cells were treated with appropriate reagents 5 min (NaIO_4_), 30 min (ConA-Cy2), or 24 h (tunicamycin) prior to infection. To verify the effect of the treatment, cells were stained with ConA-Cy2 and analysed by flow cytometry using a FACS Calibur (BD Bioscience, Heidleberg, Germany).

### Adherence assay

Cells were seeded and infected as described for gentamicin protection assays. After the infection, the cells were gently washed once, before the 293 cells were lysed by addition of 1% saponin in PBS for 15 min. Cell-associated and intracellular bacteria were suspended by vigorous pipetting, and colony forming units were determined by plating of serial dilutions on GC agar. For inhibition studies with NaIO_4_, cells were seeded in 24 well plates coated with fibronectin (4 µg/ml) with 4×10^5^ cells/well. To prevent cell detachment, cells were fixed with 4% PFA for 20 min and washed, before treatment with NaIO_4_ for 5 min at 37°C in the dark, followed by washing with PBS. After 2 h infection (MOI 40), 293 cells were lysed by addition of 1% saponin in PBS for 15 min. Bacteria attached to cells were suspended by vigorous pipetting, and colony forming units were determined by plating of serial dilutions on GC agar.

### Immunofluorescence staining

For microscopic analysis of 293 cells, 5×10^4^ cells were seeded onto poly-L-lysine- and fibronectin-coated (10 and 4 µg/ml, respectively, in PBS) glass-coverslips in 24-well plates. For microscopic analysis of A549 or HBMEC cells, 1×10^4^ cells were seeded onto 0.1% gelatine-coated coverslips in 24-well plates. Cells were infected with non-opaque (Nm Opa-) or Opa-expressing (Nm Opa_CEA_) meningococci for 2 h at an MOI of 40 (293) or an MOI of 30 (HBMEC, A549). Samples were washed once and fixed with 4% paraformaldehyde. After three washes with PBS, samples were incubated in blocking buffer (PBS, 10% fetal calf serum) for 5 min and then stained for extracellular bacteria with a polyclonal anti-Neisseria antibody (diluted 1∶100 in blocking buffer). In the case of CEACAM1-transfected 293 cells, CEACAM1 was detected by addition of monoclonal anti-CEACAM antibody (clone IH4Fc diluted 1∶100 in blocking buffer). After 1 hour, samples were washed twice, treated with blocking buffer (5 min) and incubated for 45 min with Cy5-coupled goat anti rabbit (for bacterial staining) and, if appropriate, with Cy3-coupled goat anti mouse (for CEACAM1 staining) (dilution 1∶100). Following two washes samples were incubated for 20 min with 0.1% Triton-X100 to permeabilize cellular membranes. After three washes and 5 min blocking in blocking buffer, samples were incubated with a polyclonal anti-Neisseria antibody (diluted 1∶100 in blocking buffer) for 1 hour, to detect intracellular and extracellular bacteria. Samples were washed twice, treated with blocking buffer (5 min) and incubated for 45 min with Cy2-coupled (293 cells) or Cy3-coupled (A549, HBMEC) goat anti rabbit antibody. Following three washes, samples were embedded in mounting medium (Dako, Glostrup, Denmark).

All samples were analysed with a Leica TCS SP5 confocal laser scanning microscope (Leica Microsystems, Wetzlar, Germany). Fluorescence signals of triple-labelled specimens were serially recorded with appropriate excitation and emission filters to avoid bleed-trough. Images were digitally processed with Photoshop CS (Adobe Systems, Mountain View, CA) and merged to yield pseudo-coloured pictures.

## Results

### CEACAM1 mediates association and invasion of Opa protein-negative meningococci

*Neisseria meningitidis* possesses a repertoire of virulence-associated surface structures, which promote bacterial adherence to and invasion into mammalian cells. To date, Opa_CEA_ proteins are the only neisserial proteins known to exploit members of the CEACAM-family to attach to and gain entry into the cell.

**Figure 1:**
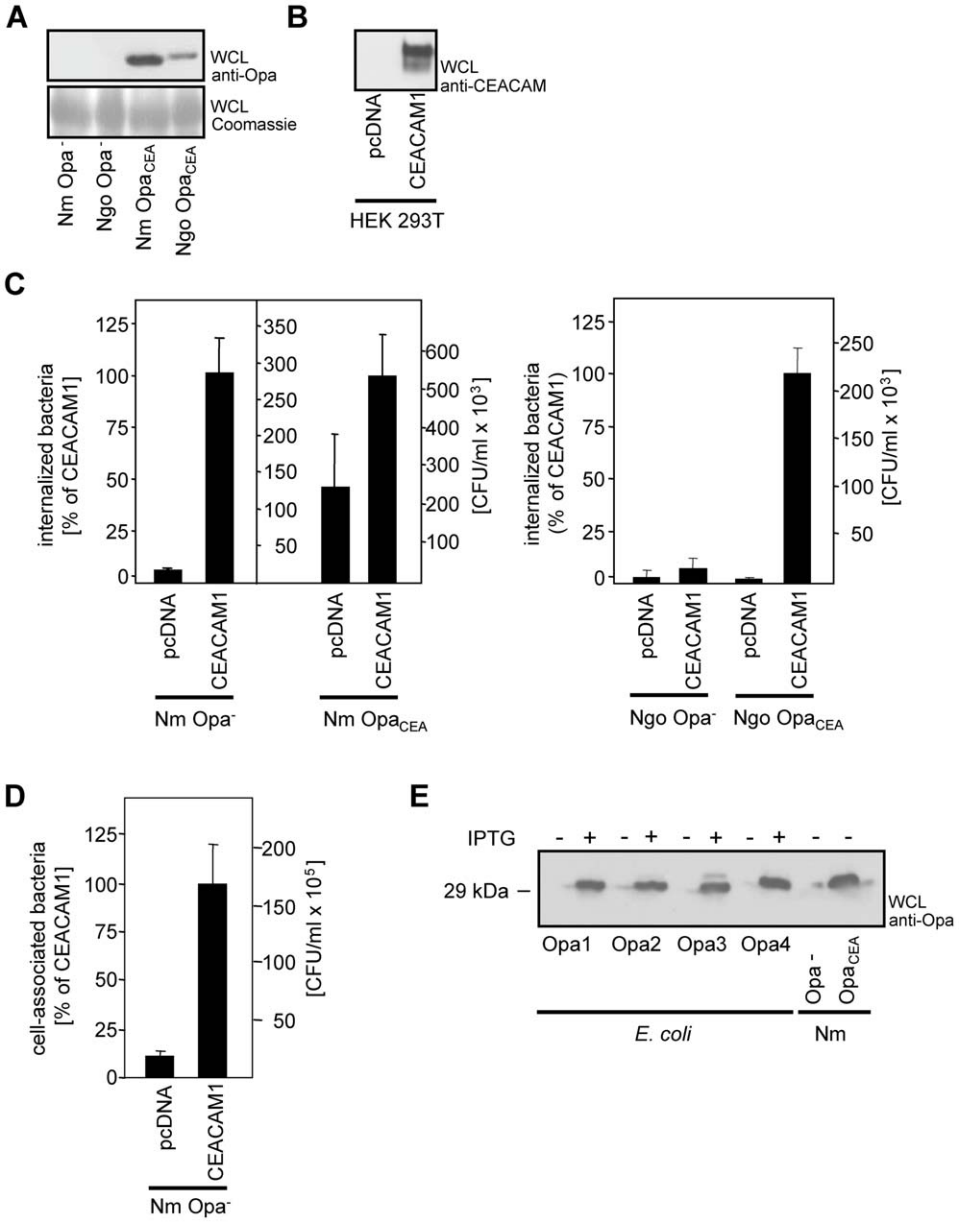
Opa protein-negative meningococci associate with and invade into CEACAM1-expressing cells. (**A**) Lysates of Opa protein-positive and Opa protein-negative *N. meningitidis* MC 58 (Nm) and *N. gonorrhoeae* MS11 (Ngo) were analysed for Opa protein expression using a monoclonal anti Opa protein-antibody. (**B**) 293 cells were transfected with an empty control vector (pcDNA) or a CEACAM1-encoding vector. Whole cell lysates (WCLs) of the transfected cells were analysed by Western blotting with a CEACAM-specific monoclonal antibody. (**C**) 293 cells as in (B) were infected for 2 h with Opa protein-negative (Nm Opa-) or Opa protein-positive meningococci (Nm Opa_CEA_) or the corresponding gonococcal strains (Ngo Opa- or Ngo Opa_CEA_, respectively). The number of internalised bacteria was determined by gentamicin protection assays. Results represent mean ± standard deviation of three independent experiments done in triplicate. Shown is the percentage of recovered bacteria compared to CEACAM1-expressing cells (left axis) as well as the absolute number of internalized colony forming units (right axis). (**D**) 293 cells as in (B) were infected for 2 h with Opa protein-negative meningococci (Nm Opa-). The number of cell-associated bacteria was determined by an adherence assay. Results represent mean ± standard deviation of one representative experiment done in triplicate. Shown is the percentage of cell-associated bacteria compared to CEACAM1-expressing cells (left axis) as well as the absolute number of cell-associated colony forming units (right axis). (**E**) The four *opa-*genes encoded in the genome of the Opa protein-negative meningococcal strain were cloned in *E. coli*. Expression of recombinant Opa1, Opa2, Opa3, or Opa4 was induced or not by IPTG and bacterial lysates were analysed for Opa protein-expression as in (A).

The current study was initiated with the aim to compare Opa_CEA_ protein-mediated host cell interaction between gonococci and meningococci. Therefore, we isolated an Opa protein-negative variant of *Neisseria meningitidis* MC58 by visual colony screening that should be used as a negative control in our studies. Indeed, the absence of Opa protein expression in this strain could be verified by Western blot using an anti-Opa protein antibody (Fig. 1A). For the invasion assay, 293 cells were transiently transfected with a CEACAM1-encoding construct or a control vector (pcDNA) and CEACAM1 expression was demonstrated by Western blotting (Fig. 1B). Two days after transfection, cells were infected with Opa protein-expressing *Neisseria meningitidis* (Nm Opa_CEA_) or *N. gonorrhoeae* (Ngo Opa_CEA_) as well as with non-opaque strains (Nm Opa-, Ngo Opa-). Following the infection, the amount of viable internalized bacteria was determined by gentamicin protection assays. As expected, Opa_CEA_ protein-expressing gonococci and meningococci were taken up by CEACAM1-expressing cells, whereas there was no invasion of non-opaque gonococci (Fig. 1C). Surprisingly, infection of CEACAM1-transfected cells with Opa protein-negative meningococci resulted in bacterial internalization into the cells. The amount of internalized Opa protein-negative meningococci (27×10^4^ cfu/ml) was lower than the amount of internalized Opa_CEA_-positive meningococci (50×10^4^ cfu/ml), but was comparable to the uptake of Opa_CEA_-positive gonococci (21×10^4^ cfu/ml) by CEACAM1-expressing cells (Fig. 1C). Clearly, control transfected cells, that did not express CEACAM1 on their surface, were not able to internalize any of the bacterial variants under these conditions demonstrating that uptake of Opa protein-negative meningococci occurs only in the presence of CEACAM1. To further analyse this interesting effect, we performed adhesion assays with Opa-negative meningococci and CEACAM1-transfected cells. In line with their ability to invade in a CEACAM1-dependent manner, the Opa protein-negative meningococci adhered to CEACAM1-expressing cells (Fig. 1D).

These results were unexpected, as Opa proteins are the only known neisserial ligands for CEACAMs. Clearly, the visually selected non-opaque strain of *N. meningitidis* MC58 did not show reactivity in Western Blots with a monoclonal α-Opa protein antibody that detects a conserved epitope in neisserial Opa proteins (Fig. 1A). However, the antigenic properties of Opa protein(s) could have changed in this strain, potentially resulting in expression of Opa proteins not recognized by the anti-Opa-antibody. To investigate, whether this is the case, all *opa* genes (*opa1*, *opa2*, *opa3*, *opa4*) encoded in the genome of this MC58-derived strain were cloned under the control of an IPTG inducible prokaryotic expression vector and overexpressed in *E. coli*. The bacterial lysates were analysed for Opa protein-expression using the same monoclonal anti-Opa-antibody. Importantly, expression of all Opa-proteins derived from *Neisseria meningitidis* MC58 (Opa1, Opa2, Opa3, or Opa4) could be detected in IPTG-induced *E. coli* strains (Fig. 1E) suggesting that the used monoclonal anti-Opa-antibody can recognize all Opa-proteins encoded in *N. meningitidis* MC58. Taken together, even though the isolated meningococcal strain does not express Opa proteins, it exploits CEACAM1 for adhesion to and invasion into the cell. Therefore, Opa-negative meningococci harbour an additional adhesin mediating interaction with CEACAM1.

### CEACAM1 – but not other members of the CEACAM family - mediates uptake of Opa protein-negative meningococci

**Figure 2:**
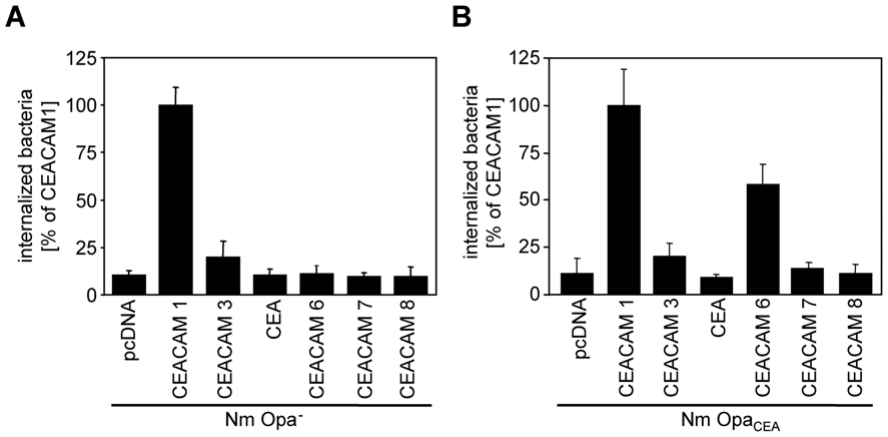
CEACAM1 – but not other members of the CEACAM family - mediates uptake of Opa protein-negative meningococci. 293 cells were transfected with constructs encoding CEACAM1, CEACAM3, CEA, CEACAM6, CEACAM7, CEACAM8 or an empty control plasmid (pcDNA). Two days after transfection cells were infected for 2 h with (**A**) Opa protein-negative meningococci (Nm Opa-) or (**B**) Opa protein-positive meningococci (Nm Opa_CEA_) and the number of internalised bacteria was determined by gentamicin protection assays. Results represent mean ± standard deviation of three independent experiments done in triplicate. Shown is the percentage of recovered bacteria compared to CEACAM1-expressing cells.

Previous results indicated that Opa proteins of pathogenic *Neisseria* can recognize several members of the CEACAM family, namely CEACAM1, CEACAM3, CEA and CEACAM6. Therefore we wondered, whether the Opa protein-negative meningococcal strain can also bind to additional members of the CEACAM-family. Accordingly, 293 cells were transfected with plasmids encoding CEACAM1, CEACAM3, CEA, CEACAM6, CEACAM7, CEACAM8 or with the empty control vector (pcDNA) and infected for two hours with either non-opaque (Nm Opa-) or Opa protein-expressing *N. meningitidis* (Nm Opa_CEA_), respectively. Gentamicin protection assays showed, that CEACAM1, but not other members of the CEACAM-family, was able to mediate internalization of Opa protein-negative meningococci (Fig. 2A). In contrast, Opa_CEA_ protein-positive meningococci were internalized by CEACAM1- and CEACAM6-expressing cells (Fig. 2B). Neither Opa-positive, nor Opa-negative meningococci were internalized by 293 cells transfected with an empty control vector (pcDNA) (Fig. 2A and B). These results suggest, that Opa-negative meningococci specifically interact with CEACAM1, but do not recognize other members of the CEACAM family.

### Microscopic analysis of CEACAM1-mediated uptake of Opa protein-negative meningococci

**Figure 3:**
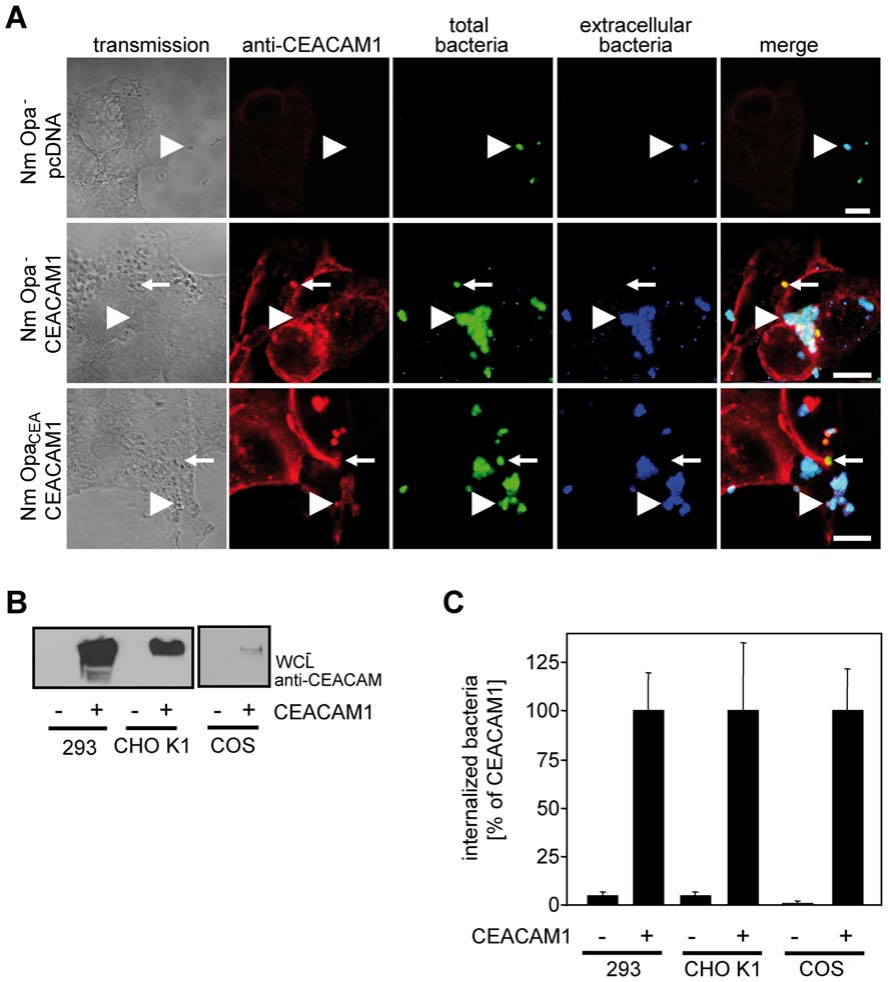
Different epithelial cell lines mediate CEACAM1-dependent internalization of Opa protein-negative meningococci. (**A**) Microscopic analysis of CEACAM1-mediated uptake in 293 cells. 293 cells were transfected with HA-tagged CEACAM1 or control vector (pcDNA). Transfected cells were infected for 2 h with Opa protein-negative (Nm Opa-) or Opa protein-expressing meningococci (Nm Opa_CEA_). Extracellular bacteria (blue) were stained using polyclonal anti-*Neisseria* antibody in combination with a Cy5-conjugated secondary antibody. After cell permeabilization, bacteria were stained again using polyclonal anti-Neisseria antibody in combination with a Cy2-conjugated secondary antibody (green) and CEACAM1 was stained using a monoclonal antibody against the HA-tag (red). Extracellular bacteria are marked by simultaneous Cy2 and Cy5 (arrowhead, blue and green), whereas intracellular bacteria are only stained by Cy2 (small arrow, green). Bars represent 10 µm. (**B**) Untransfected cells or CEACAM1-transfected 293 cells, CHO K1 cells and CHO cells were lysed and whole cell lysates (WCLs) were analysed by Western blotting using a monoclonal anti-CEACAM antibody. (**C**) CEACAM1-mediated uptake by different epithelial cells. Cells as in (B) were infected for 2 h with Opa protein-negative meningococci. The number of internalized bacteria was determined by gentamicin protection assays. Bars represent mean ± standard deviation of three independent experiments done in triplicate. Values are expressed as the percentage of internalized bacteria compared to CEACAM1.

To further confirm that CEACAM1 mediates internalization of Opa protein-negative meningococci, we performed confocal laser scanning microscopy to unambiguously detect intracellular bacteria. Accordingly, 293 cells were transfected with CEACAM1 or the empty control vector (pcDNA). Cells were infected for two hours with Opa protein-negative (Nm Opa-) or Opa protein-positive meningococci (Nm Opa_CEA_) and fixed. Samples were stained to distinguish between intracellular bacteria (stained with Cy5) and extracellular bacteria (stained with Cy2 and Cy5), and to detect CEACAM1. Confocal laser scanning microscopy revealed that both, Opa protein-positive and -negative meningococci strongly adhered to CEACAM1-expressing cells (Fig. 3A). In addition, CEACAM1-transfected cells mediated uptake of both, Opa protein-positive and Opa protein-negative meningococcal strains. As expected, control transfected cells (pcDNA) did not show association with the bacteria. Taken together, these data corroborate the findings of the adhesion assays and gentamicin protection assays and demonstrate that CEACAM1 can mediate uptake of Opa protein-negative meningococci.

### Different epithelial cell lines mediate CEACAM1-dependent internalization of Opa protein-negative meningococci

A CEACAM1-mediated uptake of Opa protein-negative *N. meningitidis* MC58 has not been observed previously, when CEACAM1-transfected CHO cells were used [46]. To exclude that internalization of non-opaque meningococci via CEACAM1 is an effect specific for transfected 293 cells, additional cell lines, such as CHO K1 cells and COS7 cells were used for CEACAM1-dependent internalization studies. Similar to 293 cells, these cells do not express any CEACAM family members endogenously. To analyze CEACAM1-mediated uptake, COS cells or CHO cells stably expressing human CEACAM1 or transfected with a control vector (Fig. 3B) were infected with Opa protein-negative meningococci at an MOI of 40. After two hours of infection, the amount of intracellular viable bacteria was determined by gentamicin protection assays. Both, CEACAM1-expressing CHO cells and CEACAM1-expressing COS cells displayed internalization of Opa protein-negative meningococci, while for control transfected cells, barely any intracellular meningococci were observed (Fig. 3C). Therefore, CEACAM1-mediated uptake of Opa protein-negative meningococci is not restricted to 293 cells and can be mediated by several CEACAM1-expressing cell lines.

### Non-opaque meningococci bind in a CEACAM1-dependent manner to cells derived from meningococcal target tissues

**Figure 4:**
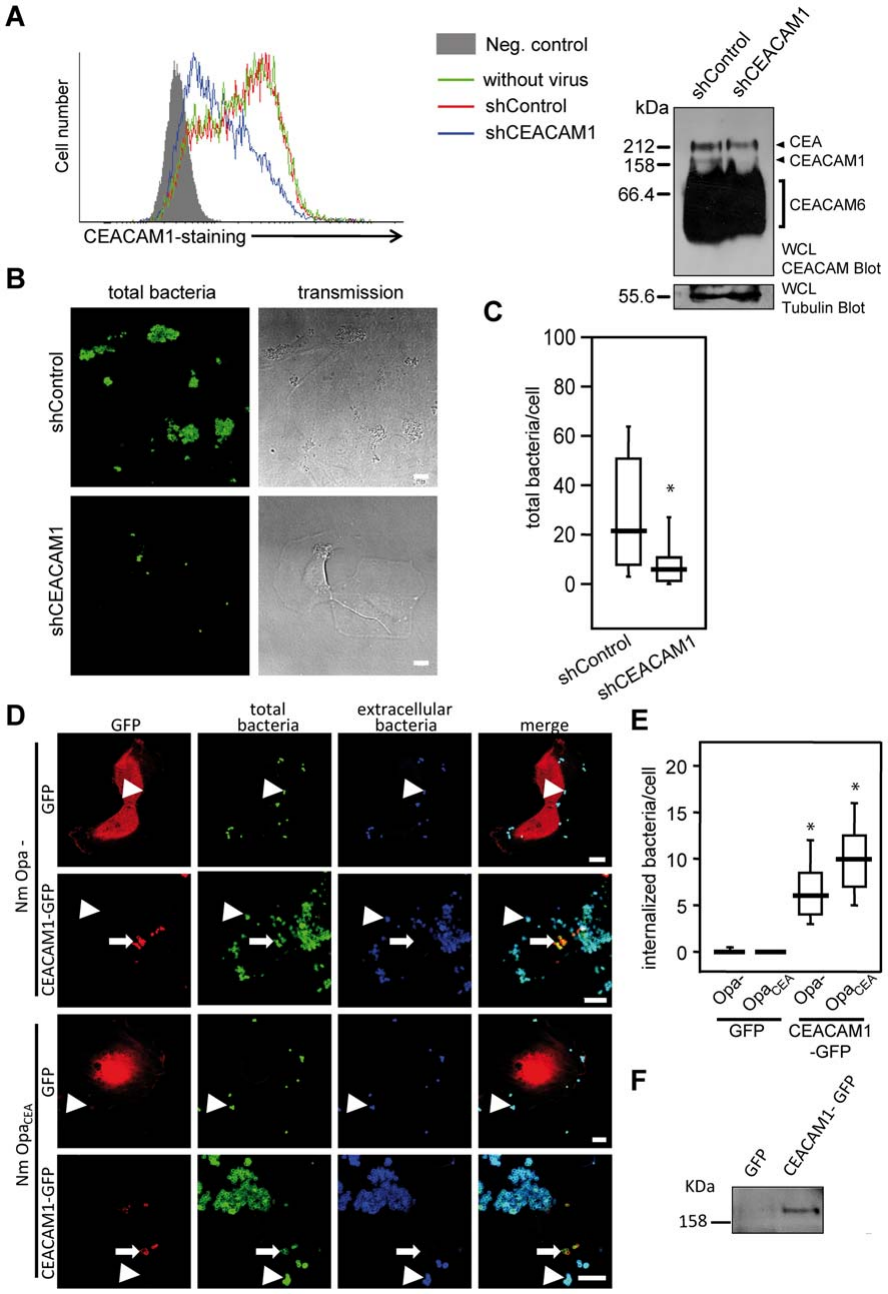
Non-opaque meningococci bind in a CEACAM1-dependent manner to cells derived from meningococcal target tissues. **A**) A549 cells were transduced with the indicated lentiviral vectors encoding an irrelevant shRNA (shControl) or a CEACAM1-targeted shRNA (shCEACAM1) or were left without virus. Transduced cells were analysed by flow cytometry with a CEACAM1/CEA-specific monoclonal antibody (clone 4/3/17). The negative control was stained with the Cy2-coupled secondary antibody only (gray curve). Whole cell lysates (WCL) of the cells were probed by Western blotting with monoclonal CEACAM-antibody (clone D14HD11; upper panel) or with monoclonal anti-tubulin antibody (lower panel). Bands derived from CEA, CEACAM1, or CEACAM6 are indicated on the right side. **B**) shControl- or shCEACAM1-transduced A549 cells were infected with non-opaque meningococci at an MOI of 30 for 3 h. Cell-associated bacteria were stained with a polyclonal antiserum (green) and cells were visualized by differential interference contrast (transmission). Bars represent 10 µm. **C**) Cell-associated bacteria were detected as in B) and counted. The line represents the median number of cell-associated bacteria and boxes represent the 25%/75% percentile from 30 cells in each sample. Groups were compared by Wilcoxon's signed rank test, * p<0.05. **D**) Human brain microvascular endothelial cells (HBMECs) were transduced with a GFP- or a CEACAM1-GFP encoding lentivirus. Transduced cell populations were infected with Opa_CEA_-expressing (Nme Opa_CEA_) or non-opaque (Nme Opa-) *N. meningitidis* at an MOI of 30 for 3 h. Samples were differentially stained with polyclonal antiserum against *N. meningitidis* to differentiate extracellular (arrowhead) and intracellular (small arrow) bacteria. Transduced cells are detected by their GFP signal. Bars represent 10 µm. **E**) Samples from D) were enumerated for intracellular meningococci as in C). **F**) Whole cell lysates (WCL) of virus transduced cells from D) were probed with an anti-GFP antibody to demonstrate expression of CEACAM1-GFP.

Meningococci colonize the mucosal surface of the nasopharynx and can interact with endothelial cells of the blood-brain barrier during meningococcal disease [4]. To test, if Opa protein-negative meningococci are able to interact with CEACAM1 present on target tissues, we employed the adenocarcinoma cell line A549 derived from lung alveolar epithelium. These cells endogenously express CEACAM1, CEA and CEACAM6 [47] (Fig. 4A). To modulate CEACAM1 levels in these cells, we transduced A549 cells with a lentiviral vector encoding short hairpin RNA (shRNA) directed against human CEACAM1 (shCEACAM1). Indeed, CEACAM1 expression was suppressed by more than 50% in the shCEACAM1 cells compared to untransduced cells or compared to A549 cells transduced with a non-targeted, irrelevant shRNA (shControl) (Fig. 4A; left panel). Despite the remaining presence of CEA and large amounts of CEACAM6 (Fig. 4A; right panel), knockdown of CEACAM1 in shCEACAM1-transduced A549 cells led to a significantly reduced cell association by non-opaque meningococci (Fig. 4B and C).

To further analyse the role of CEACAM1 recognition for target cell interaction, we employed human microvascular endothelial cells (HBMECs). Unstimulated primary endothelial cells do not express CEACAMs, but a strong upregulation of CEACAM1 is observed upon stimulation with pro-inflammatory cytokines such as TNFα, which is induced during meningococcal infection [48]. Accordingly, we transduced HBMECs with a lentiviral vector encoding CEACAM1-GFP or a control virus encoding GFP. Endothelial cells were infected with opaque or non-opaque meningococci and the cell association and internalization was evaluated by immunofluorescence microscopy (Fig. 4D). Both cell-association and internalization of meningococci were strongly increased upon CEACAM1 expression and this was true for Opa_CEA_ protein-expressing and non-opaque bacterial variants (Fig. 4D and E). Internalization of meningococci occurred to a significant degree only in cells expressing CEACAM1, again demonstrating that similar to Opa_CEA_ protein-expressing pathogens, non-opaque *N. meningitidis* can interact with target cells in a CEACAM1-dependent manner (Fig. 4D–F).

### CEACAM1-mediated uptake of Opa protein-negative meningococci is independent of carbohydrate moieties

**Figure 5:**
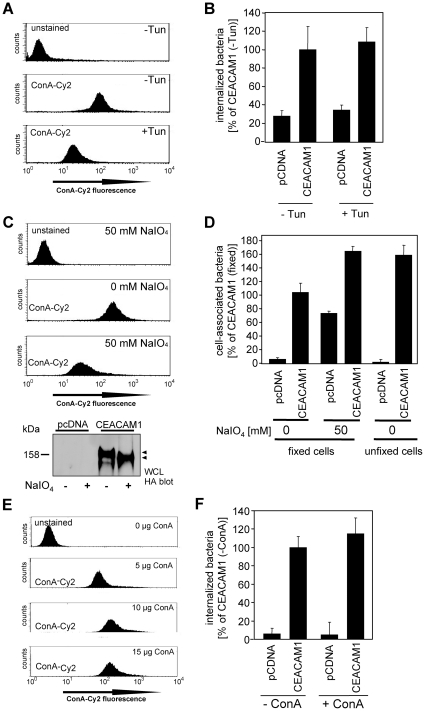
CEACAM1-mediated uptake of non-opaque meningococci is independent of receptor glycosylation. (**A–F**) 293 cells were transfected with an empty control vector (pcDNA) or HA-tagged CEACAM1. (**A, B**) Cells were treated with tunicamicin (+Tun; 2 µg/ml) 24 h prior to infection or were left untreated (-Tun). A) Cells were stained with concanavalin-Cy2 (ConA-Cy2) to label surface glycoproteins or were left unstained and then analysed by flow cytometry. B) Cells were infected with Opa-negative meningococci and the number of internalized bacteria was determined by gentamicin protection assays. Results represent mean ± standard deviation of three independent experiments done in triplicate and show the percentage of recovered bacteria in comparison to CEACAM1-expressing cells in the absence of Tunicamicin. (**C, D**) Transfected 293 cells were fixed and incubated with NaIO_4_ (50 mM) for 5 min at 37°C in the dark or were left untreated. As a further control, unfixed cells, which were not treated with NaIO_4_, were used. C) Cells were analysed for surface glycoproteins as in A) and whole cell lysates (WCL) of pcDNA or CEACAM1-HA transfected cells with or without NaIO_4_ treatment were probed with anti-HA-tag antibodies. The size change upon NaIO_4_ treatment is indicated by arrowheads. D) Cells were infected with Opa protein-negative meningococci and the number of internalized bacteria was determined as in B). (**E, F**) Transfected 293 cells were incubated with the indicated concentrations of concanavalin-Cy2 (ConA-Cy2) to saturate α-mannopyranosyl- and α-glucopyranosyl residues. E) Cells were analysed by flow cytometry. F) Cells were treated (+ConA) or not (-ConA) with 15 µg ConA-Cy2, infected with Opa protein-negative meningococci and employed in gentamicin protection assays as in B).

CEACAM1 is a highly glycosylated receptor. Whereas pathogens such as Opa_CEA_ protein-expressing *Neisseriae*, *Moraxella catarrhalis*, or *Haemophilus influenzae* recognize protein determinants of CEACAM1 [49], [50], [51], [52], several enterobacteria associate with carbohydrate moieties of the receptor [53], [54]. As Opa protein-negative meningococci must employ a novel adhesin for CEACAM1 binding, we were interested whether glycosylation of CEACAM1 is essential for this interaction. To influence the level of receptor glycosylation, CEACAM1 transfected cells or control transfected cells were pre-treated with tunicamycin, an inhibitor of N-glycosylation, or NaIO_4_, which mediates oxidation of carbohydrate cis-diol-groups to aldehyds. Untreated cells served as a negative control. The decrease in surface glycosylation after pre-treatment was investigated by flow cytometry using the Cy2-labeled plant lectin concanavalin A (ConA-Cy2), which recognizes α-mannopyranosyl- and α-glucopyranosyl-residues. Indeed, pre-treatment of cells with tunicamycin (2 µg per 4×10^5^ cells) as well as treatment of cells with the oxidant NaIO_4_ (50 mM) strongly reduced the presence of ConA-binding carbohydrate moieties (Fig. 5A and C). Furthermore, NaIO_4_ treatment increased the mobility of CEACAM1 upon SDS-gel electrophoresis demonstrating the successful oxidation of terminal carbohydrate residues (Fig. 5C). However, treatment of CEACAM1-expressing cells with tunicamicin or NaIO_4_ did not influence binding and internalization of Opa protein-negative meningococci (Fig. 5B and D). As observed before, there was no internalization in control transfected cells (Fig. 5B and D). To further analyse the involvement of carbohydrate moieties in CEACAM1 mediated uptake, we blocked surface-located α-mannopyranosyl- and α-glucopyranosyl-residues by preincubation of CEACAM1-transfected cells with ConA-Cy2 at a concentration of 15 µg/1.5×10^6^ cells. This concentration was sufficient to saturate all ConA-interacting carbohydrate-structures located on the surface of the cells (Fig. 5E). Again, blocking of surface-located α-mannopyranosyl- and α-glucopyranosyl-residues did not influence CEACAM1-mediated uptake of Opa protein-negative meningococci (Fig. 5F). Together, these data suggest, that CEACAM1-mediated uptake of Opa protein-negative meningococci does not involve carbohydrate structures of the receptor.

### CEACAM1-mediated uptake of Opa protein-negative meningococci requires several extracellular domains of the receptor

**Figure 6:**
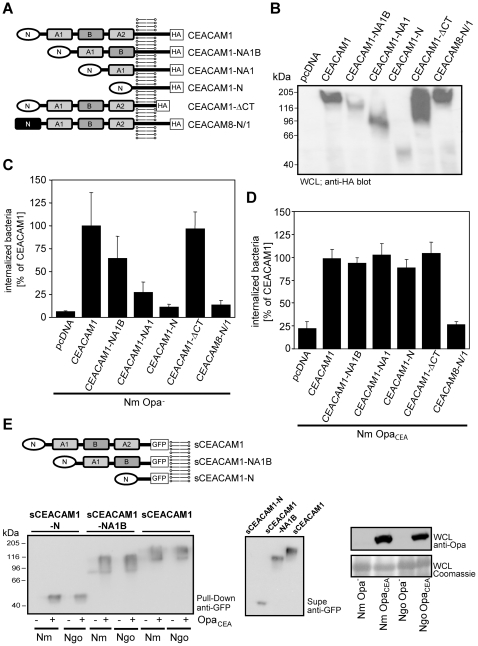
CEACAM1-mediated uptake of Opa-negative meningococci requires multiple extracellular immunoglobulin domains. (**A**) Schematic drawing of CEACAM1, CEACAM1-mutants and the CEACAM8/CEACAM1 chimera. CEACAM1-mutants (CEA1-NA1B, CEA1-NA1, CEA1-N) are composed of an amino-terminal Ig_V_-like domain (N) and a variable number of Ig_C2_-like domains (A1, B, or A2). The CEA1-ΔCT mutant lacks the cytoplasmic domain and in the CEACAM8/CEACAM1 chimera (CEA8N/1) the Ig_V_-like, amino-terminal domain of CEACAM1 is exchanged with the corresponding domain of CEACAM8. All constructs harbour a carboxy-terminal HA-tag. (**B**) Expression of CEACAM1, CEACAM1-mutants and CEA8N/1 in whole cell lysates (WCLs) of transfected 293 cells is analysed by Western blotting with a monoclonal anti-HA-tag antibody. (**C, D**) 293 cells transfected with the indicated constructs were infected for 2 h with C) Opa protein-negative meningococci (Nm Opa-) or D) with Opa protein-positive meningococci (Nm Opa_CEA_). Internalised bacteria were determined by gentamicin protection assays. Results represent mean ± standard deviation of three independent experiments done in triplicate and show the percentage of internalized bacteria compared to CEACAM1. (**E**) The indicated soluble GFP-tagged CEACAM1 variants were produced in 293 cells and the cell culture supernatants were used in pull-down assays with Opa protein-negative (Opa-) or Opa protein-positive (Opa_CEA_) meningococci (Nm) or gonococci (Ngo). Precipitates were probed with polyclonal anti-GFP antibody to detect bacteria-associated CEACAM1 (left panel). The presence of equal amounts of the sCEACAM1 variants in the supernatants (Supe) was verified by blotting with GFP-antibodies (middle panel). The Opa phenotype of the used bacteria was analysed by Western blotting with monoclonal anti- Opa protein antibody (right panel).

Human CEACAM1 is a receptor recognized by diverse adhesins of several bacterial pathogens [33]. For Opa protein-mediated interactions, the amino-terminal domain of CEACAM1 is necessary and sufficient for binding. Therefore, we were interested, which of the extracellular domains of the receptor were needed for association with the putative CEACAM1-binding adhesin of Opa-negative meningococci. Accordingly, we generated a panel of CEACAM1-constructs with a varying number of extracellular domains (Fig. 6A). The constructs encompassed the Ig_V_-like amino-terminal domain (N) of CEACAM1, followed by none (CEACAM1-N), one (CEACAM1-NA1), or two (CEACAM1-NA1B) Ig_C2_-like extracellular domains of the receptor, fused to the transmembrane and cytoplasmic domain. In addition, a chimeric protein composed of the amino-terminal domain of CEACAM8 – which does not interact with any CEACAM-binding adhesin or with Opa protein-negative meningococci – followed by the Ig_C2_-like, transmembrane and cytoplasmic domain of CEACAM1 (CEACAM8-N/1). All constructs contained a carboxy-terminal HA-tag for detection. Similar to wildtype CEACAM1 and CEACAM1 lacking the cytoplasmic domain (CEACAM1-ΔCT) these constructs were expressed in 293 cells with the expected size (Fig. 6B). Gentamicin protection assays revealed that neither Opa protein-positive, nor Opa protein-negative meningococci were internalized by the CEACAM8-N/1-chimeric protein, demonstrating the importance of the amino-terminal domain of CEACAM1 for bacterial internalization in both cases (Fig. 6C and D). However, the CEACAM1 mutants with deletions in the extracellular Ig_C2_ domains showed a distinct pattern of interaction with either Opa protein-positive or Opa protein-negative *N. meningitidis*. Whereas opaque meningococci were internalized to the same extent by all CEACAM1 constructs harbouring the amino-terminal domain of CEACAM1 (CEACAM1, CEACAM1-NA1B-variant, CEACAM1-NA1-variant, CEACAM1-N-variant, CEACAM1-ΔCT), internalization of Opa protein-negative meningococci was strongly affected by a reduction in the number of Ig_C2_-like extracellular domains of CEACAM1 (Fig. 6C and D). These data demonstrate that optimal CEACAM1-mediated invasion of Opa protein-negative meningococci seems to depend on multiple extracellular domains of this receptor. The amino-terminal domain of CEACAM1 is necessary (no invasion via CEACAM8-N/1-chimera), but not sufficient (no invasion via CEACAM1-N-variant) for efficient uptake of Opa protein-negative meningococci.

### Soluble CEACAM1 extracellular domains do not bind to non-oapque meningococci

CEACAM1 forms parallel cis-dimers in the membrane that are supported by the extracellular Ig_C2_-like domains [55]. To test, if such a particular tertiary and quaternary structure of CEACAM1 might be required to allow binding of Opa protein-negative meningococci, soluble forms of CEACAM1 (sCEACAM1) that encompass either one or multiple extracellular domains were generated (Fig. 6E). These proteins were expressed in 293 cells and the cell-free supernatants were used in pull-down analyses with either non-opaque or Opa_CEA_ protein-expressing meningococci or the corresponding gonococcal variants. As expected, both Opa_CEA_ protein expressing neisserial strains associated with all soluble CEACAM1 variants (Fig. 6E). However, neither non-opaque gonococci nor non-opaque meningococci were able to precipitate any of the CEACAM1 proteins in this assay format (Fig. 6E). These results are in agreement with previous analyses using non-opaque meningococci or gonococci together with soluble CEACAM1 receptor domains [35]. Furthermore, these results indicate that the overall tertiary and/or quaternary structure of CEACAM1 on the cell surface is critical to provide a binding interface for non-opaque meningococci.

### Known meningococcal invasins do not contribute to CEACAM1-mediated internalization of non-opaque meningococci

Several adhesive proteins of meningococci have been identified [11]. Whereas type IV pili, App, MspA, HrpA and NhhA promote adherence to host cells, Opa proteins, Opc and NadA have been shown to additionally trigger entry into mammalian cells and to function as invasins. In a candidate approach to elucidate the CEACAM1-directed invasin of non-opaque meningococci, we analysed the contribution of NadA and Opc in this internalization process.

**Figure 7:**
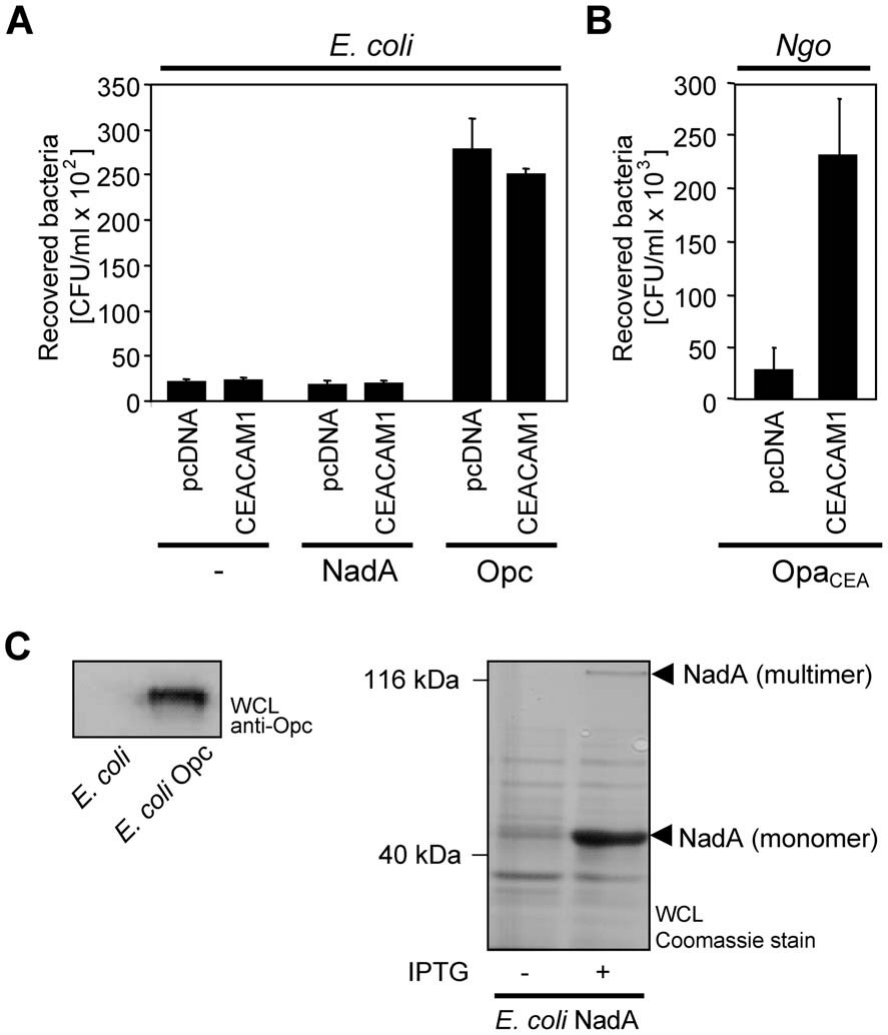
Known meningococcal invasins do not contribute to CEACAM1-mediated internalization of non-opaque meningococci. (**A**) 293 cells were transfected with pcDNA CEACAM1-4L-HA or with the empty control vector (pcDNA). Two days after transfection, cells were infected for 2 h with *E. coli* strains recombinantly expressing NadA, Opc, or the parent strain. The number of internalised bacteria was determined by gentamicin protection assays. Results represent mean ± standard deviation of three independent experiments done in triplicate and show the percentage of recovered bacteria compared to CEACAM1. (**B**) Cells as in (A) were infected with Opa_CEA_-expressing gonococci (Ngo Opa_CEA_) and the number of internalised bacteria was determined as in (A). (**C**) Expression of recombinant NadA and Opc in *E. coli*. Expression of Opc was analysed in bacterial lysates by Western Blot using monoclonal anti-Opc antibody. Expression of recombinant NadA in *E. coli* was analysed by SDS-PAGE after induction with IPTG. The multimeric, functional form of NadA is indicated (arrow). As a negative control, non-induced *E. coli* or *E. coli* not transformed with recombinant proteins were used.

Therefore, CEACAM1 transfected cells or control transfected cells were infected for two hours with *E. coli* expressing either NadA or Opc or the parental *E. coli* strain at an MOI of 40. Bacterial uptake was then evaluated by gentamicin protection assays (Fig. 7A). Cells infected with Opa_CEA_ protein-expressing gonococci served as a positive control (Fig. 7B). The expression of the invasins in the *E. coli* strains was confirmed by Western blot (Opc) or by SDS-PAGE (NadA) (Fig. 7C). As expected, infection of CEACAM1-transfected cells with Opa_CEA_ protein-expressing bacteria resulted in efficient internalization (Fig. 7B). Infection of 293 cells with *E. coli* NadA or the *E. coli* control strain did not result in bacterial internalization and this was unchanged upon CEACAM1 expression (Fig. 7A), suggesting that the NadA receptor is not present in 293 cells and that CEACAM1 is not recognized by this bacterial invasin. In contrast, Opc-expressing *E. coli* invaded control cells as well as CEACAM1-expressing cells at similar levels (Fig. 7A). Clearly, the lack of increased invasion of Opc *E. coli* into CEACAM1-expressing cells indicates that this invasin is not involved in CEACAM1-mediated uptake. Therefore, these data suggest that *N. meningitidis* MC58 expresses a novel, undescribed invasin, which utilizes CEACAM1 as a cellular receptor to gain entry into mammalian cells.

## Discussion

Bacterial adhesion to epithelial cells is of prime importance in mucosal colonization. However, binding of bacterial adhesins to host receptor molecules may also lead to tissue invasion, a likely pre-requisite for dissemination. In the case of *N. meningitidis*, an array of outer membrane molecules, such as Opc, NadA and Opa proteins can promote bacterial invasion into host cells [11]. Opa proteins bind to members of the CEACAM family and have been the only known neisserial ligands for these cellular receptors [32]. In the current study we performed invasion studies with an Opa protein-negative derivative of the meningococcal strain *N. meningitidis* MC58. Surprisingly, we found that this strain was able to bind to and invade human cells in a CEACAM-dependent manner. Opa protein-negative *N. meningitidis* specifically interacted with CEACAM1, but not with other members of the CEACAM family. Hence, we postulate that this Opa protein-negative meningococcal strain must possess a second, CEACAM1-binding invasin, distinct from Opa proteins.

Previously, we have investigated receptor recognition by Opa protein-expressing and Opa protein-negative *N. meningitidis* using the soluble amino-terminal Ig_V_-like domains of various human CEACAMs including CEACAM1 [35]. In these former studies, we did not observe an interaction of Opa protein-negative meningococci with CEACAM1. However, the fact that receptor engagement by the second CEACAM1-binding meningococcal adhesin requires multiple extracellular domains of CEACAM1 explains our previous negative results. In addition, other scientists examined the interaction of an Opa protein-negative strain of *N. meningitidis* MC58 with CEACAM1 by performing receptor overlay assays with a recombinant soluble molecule. Also in this case, they could not detect any association of Opa-negative meningococci with CEACAM1 in this assay format, though the used construct encompassed the Ig_C2_-like extracellular domains of CEACAM1 [46]. Since in our analysis CEACAM1-binding by Opa protein-negative meningococci occurs in a cellular context, but not with recombinant, soluble CEACAM1 proteins, one might speculate that the overall presentation of CEACAM1 on the cell surface is different from the conformation of the soluble molecules. Indeed, recent structural investigations have detected the assembly of parallel cis-dimers of CEACAM1 that involve not only the amino-terminal Ig_V_-like domain, but that are supported by the extracellular Ig_C2_-like domains [55]. Therefore, a significant fraction of membrane-bound wildtype CEACAM1 is present in the form of cis-dimers that due to allosteric interactions might expose a binding interface different from soluble recombinant CEACAM1.

A second, mutually non-exclusive explanation for the previous failure to detect Opa-independent interactions with CEACAM1 could be that the second CEACAM1 binding adhesin is subject to phase variation and might be incidentally in an “on”-state in the Opa protein-negative strain used in our study. In *Neisseriae*, phase variation can occur through several mechanisms including alterations in the number of short sequence repeats in the coding region of genes or through variation in poly-nucleotide stretches in promoter regions [56], [57]. The hypothesis, that the second CEACAM1-binding adhesin is subject to phase variation, is further strengthened by the finding that the genome of *Neisseria meningitidis* MC58 encodes, with more than one hundred candidates, one of the largest known repertoires of phase variable genes [58]. Clearly, several neisserial virulence factors, such as pili, capsule, lipooligosaccharide, Opc and Opa proteins, are expressed in a phase variable manner, which possibly enhances the capacity of the organism to successfully colonize its narrow ecological niche and to evade the host immune response.

To date, several CEACAM1 targeting ligands from diverse pathogens have been described, such as protein P5 (*Haemophilus influenzae*), UspA1 (*Moraxella catarrhalis*), AfaE/DraE adhesins (*Escherichia coli*), and Opa_CEA_ proteins (pathogenic *Neisseria*) [22], [23], [52], [59], [60], [61]. Though these adhesins target overlapping binding sites on the non-glycosylated face of the aminoterminal Ig_V_-like domain, they are structurally highly diverse [50], [51], [52]. Whereas protein P5 and Opa_CEA_ proteins form transmembrane β-barrel structures with four surface exposed extracellular loops [62], [63], [64], the trimeric autotransporter protein UspA1 has a lollipop structure consisting of a head group, an extended coiled-coil stalk region, and a membrane anchor domain [65]. Because of this conserved structural organisation, which is similar to meningococcal NadA, UspA1 belongs to the Oca family [66]. The prototype of this adhesin family, YadA from enteropathogenic *Yersiniae*, binds to extracellular matrix proteins via determinants in its head domain [67]. Though UspA1 can also bind to extracellular matrix proteins via its head domain, the CEACAM-binding site of UspA1 is located at a distant site in the trimeric coiled-coil stalk region [52], [68]. By sequence comparison of CEACAM1-associating and non-associating variants of UspA1, the binding site has been narrowed down to a short linear region of ∼70 amino acids [69]. Interestingly, a 10 amino acid stretch of this sequence is also present in the stalk of *N. meningitidis* NadA. However, we did not observe CEACAM1 binding by recombinant *E. coli* expressing the oligomeric, functional form of this protein suggesting that NadA is not the second CEACAM1-binding adhesin of *N. meningitidis*.

Similar to Opa protein-mediated interactions, we could demonstrate that binding of non-opaque meningococci to CEACAM1 is independent of carbohydrate moieties of the receptor. Furthermore, the aminoterminal domain of CEACAM1 is necessary for association with both Opa_CEA_ protein-expressing and Opa protein-negative meningococci. However, in contrast to Opa_CEA_ protein-expressing *Neisseriae* or UspA1 of *Moraxella catarrhalis*, the aminoterminal domain alone is not sufficient for internalization of non-opaque meningococci, as efficient uptake requires several extracellular domains of CEACAM1. This is reminiscent of the situation with typeable and non-typeable *H. influenzae*, where in most cases the aminoterminal Ig_V_-like domain of CEACAM1 is necessary, but not sufficient for binding, which requires additional Ig_C2_-domains [51]. Though the exact CEACAM1-binding determinants of P5 have not been elucidated, the protein-protein-interaction between the *Haemophilus* adhesin and its host receptor is sensitive to mild denaturation [51] suggesting again that a specific tertiary or quaternary structure of the complete extracellular domain of CEACAM1 is critical.

The expression of multiple, independent ligands for CEACAM1 suggests that targeting of this receptor may be a key element in colonization and pathogenesis of *N. meningitidis*. Interestingly, engagement of CEACAM1 by meningococci can enhance the matrix adhesion of infected host cells, a process that could counteract the exfoliation of superficial cells in a squamous or stratified epithelial tissue [38]. Indeed, it has been shown recently that engagement of CEA on the mucosal surface of the murine urogenital tract interferes with the exfoliation of superficial epithelial cells, thereby promoting bacterial colonization of this tissue [43]. This process depends on the upregulation of an additional host protein, CD105, which promotes integrin activation and increased adhesion of the infected epithelial cells to the extracellular matrix [43]. As CD105 upregulation is also induced in human epithelial cells upon stimulation of CEACAM1 by CEACAM binding Neisseria [38], the presence of several CEACAM1-binding adhesins might be highly advantagous during the initial host colonization. Furthermore, an alternative, Opa protein-independent system for CEACAM1-binding could allow the bacteria to exploit the immunosuppressive functions of CEACAM1 by arresting T-cell proliferation *in* vitro, even when Opa protein expression is modulated due to phase variation [70].

In the current study we have provided evidence that *Neisseria meningitidis* MC58 possesses a second, CEACAM1-binding invasin. As this factor mediates cell-adhesion and host cell invasion in the absence of Opa proteins and by a mechanism distinct from these well-known meningococcal outer membrane proteins, our study provides impetus to further characterize and identify this novel invasin.
